# Depression, Depressive Somatic or Nonsomatic Symptoms, and Function in a Primarily Hispanic Chronic Pain Population

**DOI:** 10.1155/2013/401732

**Published:** 2012-12-01

**Authors:** Kristynia M. Robinson, Jose J. Monsivais

**Affiliations:** ^1^School of Nursing, The University of Texas at El Paso (UTEP), 1851 Wiggins Road, Room 338, El Paso, TX 79968, USA; ^2^Paul L. Foster School of Medicine, Texas Tech Health Science Center (TTHSC), Hand and Microsurgery Center of El Paso, 10175 Gateway Boulevard W, No. 230, El Paso, TX 79925, USA

## Abstract

Chronic pain and depression are two major causes of disability. Comorbidity decreases psychosocial and physical functioning while increasing economic burden. The prevailing belief that Hispanics somaticize depression may hinder the diagnostic process and, thus, may impact outcomes. The purpose of this study was to explore the relationships among depression and depressive symptoms (somatic or nonsomatic) and function in chronic pain sufferers residing along the USA-Mexico border. Like other studies, as level of depression increased, level of pain increased and level of functioning decreased. So much so that almost a quarter of the participants reported moderate-to-severe depression, and another quarter of the participants reported suicidal ideation independent of depression or treatment. Unlike other published reports, we used a sample of chronic pain patients who received individualized, multimodal pain treatment. Compared to our previous work in a similar population, pain intensity and suicidal ideation were lower in this study. A plausible explanation is the use of antidepressants as adjuvant treatment for pain. Regardless of gender or ethnicity, persons with chronic pain will disclose symptoms of depression when appropriate tools are used to collect the data. Implications for future research and clinical practice are discussed.

## 1. Introduction

Chronic pain and depression are similar in many ways. These conditions share common neurological pathways [1–3] and remain underdiagnosed and undertreated leading to decreased functioning and poor clinical outcomes [4]. As contested illnesses, they defy the mind-body dualism present in allopathic medicine, lack visible pathology and are subjective in nature [5–7]. Often invisible pathology and subjective complaints are delegitimized [8, 9] leaving individuals feeling stigmatized [6, 10–12]. Consequences of both conditions include intra- and interpersonal conflicts, cognitive complaints, social impairment, and financial distress [2, 13]. Furthermore, disability and economic burden in terms of healthcare costs and lost productivity are additive with comorbidity [4, 14].

Depressive symptoms may be masked or overlooked when the reason for a health care visit is chronic pain, especially if a depressed individual presents with somatic (physical) symptoms. Diagnosis of comorbid depression may be impacted by a prevailing, perhaps unfounded, belief that Hispanics tend to somaticize depression [15–18], that is, express mental distress as physical complaints [19, 20] and, therefore, are more likely to seek healthcare for physical than emotional distress. Yet, research is sparse in comorbidity studies of depression and pain in Hispanics.

## 2. Background

Chronic pain and depression are two major causes of disability [4, 22]. Prevalence is high in the general population. Approximately one in three Americans suffers chronic pain [22] and one in five experiences depression during their lifetime [23]. Chronic pain and its personal, physiological, and social impact are nondiscriminatory in the general population. Comorbid somatic symptoms of pain and discomfort accompany depression about 50%–60% of time [4, 24, 25]. Although Hispanics tend to be low risk for depression [23, 26], the prevalence of depression in chronic pain tends to be the same regardless of ethnicity [16, 24, 27, 28]. 

Physiologically, pain and depression share common neurological pathways and impact the same neurotransmitters [1–3]. Individuals with pain often develop depression [4, 25], and individuals with depression have poorer outcomes when they experience pain [29]. Some researchers suggest that disability is greater and physical functioning worse in individuals with pain who somaticize depression [16, 20].

The population for this study is located in a southwestern border community that is primarily Hispanic (82%) and poor, both of which contribute to health disparities [30]. Compared to the Anglo population, the Hispanic population has significantly worse physical health outcomes [31, 32]. In 2010, over 25% of Hispanics in this community reported their general health to be fair or poor compared to 16.5% of the non-Hispanic white population [33]. Therefore, studying pain and depression, two common causes of disability, in a primarily Hispanic border population is warranted.

## 3. Purpose

Research related to pain and depression is sparse in Hispanics of Mexican origin. Therefore, the purpose of this cross-sectional study was to explore the relationships among depression and depressive symptoms (i.e., somatic or nonsomatic) and function in a population of chronic pain sufferers residing along the USA-Mexico border in far west Texas. Minimum sample size was set at 85 based on *a priori* power analysis to detect a medium size effect (relationship) between psychosocial variables of pain, function, and depression (*α* of 0.05 with power of 0.80) [34]. 

## 4. Methodology

This research is part of an exploratory study on acculturation, depression, and function in individuals seeking pain management in a predominantly Hispanic border community [35]. Data were collected during the fall of 2006 and spring of 2007. University policies and procedures were observed to protect human rights and HIPAA regulations followed to ensure privacy. We received Institutional Review Board approval from The University of Texas at El Paso prior to data collection. 

Written informed consent in primary language spoken by the participant was obtained prior to being seen by the physician or nurse practitioner. Bilingual staff assisted low-literacy participants by reading out loud the consent form. Coercion was prevented by blinding authors to patient participation. To maintain confidentiality and anonymity data were deidentified and reported in aggregate form.

## 5. Setting

We recruited participants in the private practice of the second author, a hand and microsurgery specialist board certified in pain management by the American Board of Pain Medicine. About half of the patients seen at the clinic are treated for chronic pain. Clinic staff is multicultural and bilingual (English/Spanish). The chronic pain patient population of this clinic mirrors that of the southwestern border community of El Paso in that patients are predominantly Hispanic of Mexican origin whose primary language is Spanish. 

## 6. Sample

For this study, we used convenience sampling and oversampled to optimize recruitment and retention of 85 participants. Clinic staff approached the first 100 unduplicated pain patients seen in the clinic after the start of the study. Seven patients did not meet inclusion criteria; the remaining patients who met inclusion criteria agreed to participate in the study. Inclusion criteria included English or Spanish speaking adults 18 years or older who resided along the USA-Mexico border, sought treatment for chronic pain, and were able to give informed consent. Once the consent form was signed, participants completed demographic information and scales for pain, depression, and function in their primary language prior to seeing the provider. If needed, a trained staff person assisted the individual with completion of the forms. Participants received $5.00 as compensation for time.

## 7. Measures

### 7.1. Demographic Data

We developed a form to collect demographic data relevant to the population under study. Data collected included self-reported gender and ethnicity, year and country of birth, level and place of education (Mexico or United States), occupation, and annual income. Physiologic and diagnostic data, such as body mass index (BMI), diagnoses (ICD-9 codes), and treatment regimen were extracted from medical record after enrollment in study. 

### 7.2. Pain

For this study, we defined chronic pain as pain persisting three months or longer, a definition consistent with that of the International Association for the Study of Pain (IASP) [36]. Pain intensity and interference with life (psychosocial and physical functioning) were measured using the Brief Pain Inventory (BPI) short form, a multidimensional pain assessment scale [37]. The BPI short form takes about 5 minutes to complete. 

The BPI consists of 9 items including four equidistant 0–10 numerical rating scales (NRSs) (from no pain to worst pain imaginable) for “right now” or current and worst, average, and least pain intensity over the past 24 hours. Four separate BPI items inquire about (a) presence of pain today, (b) location of pain, (c) current pain treatments or medications, and (d) pain relief in past 24 hours attributed to current pain treatments or medications. The last (question 9a–g) measures interference with seven domains of function—general activity, mood, walking, work, relationships, sleep, and enjoyment of life—using an equidistant 0–10 NRS (from no to complete interference in past 24 hours) [37]. Numerous researchers report high reliability and strong validity for English and Spanish versions of the BPI in chronic nonmalignant pain populations [38, 39].

### 7.3. Function

For this study, function is defined as the psychosocial and physical ability to conduct daily activities essential to living a productive and enjoyable life. To capture function we used BPI question 9 that provided a snapshot of pain interference on function over past 24 hours and the SF-36 short form version 2 (SF-36vr2) that measured quality of life and overall functioning over the past 4 weeks [40]. The SF-36vr2 survey takes approximately 20–30 minutes to complete.

The SF-36vr2 provides summary scores for physical (physical component summary (PCS)) and mental (mental component summary (MSC)) functioning. Physical functioning encompasses subscales of physical functioning, role-physical, bodily pain, and general health while mental functioning covers vitality, social functioning, role-emotional, and mental health. Norm-based scoring (0–100) allows comparison across general and specific adult populations; for example, anything less than 50 on the SF-36vr2 indicates worse functioning than the average American [40]. Demonstrated reliability and validity are reported for English and Spanish versions [41].

### 7.4. Depression and Somatic/Nonsomatic Symptomatology

For this study, depression is defined as decreased pleasure in life [42]. Depressed individuals may present with somatic (physical), nonsomatic (psychosocial or behavioral), or a combination of symptoms. Overall depression and somatic/nonsomatic symptomatology were measured using the Beck Depression Inventory-II (BDI-II) [21]. The BDI-II takes about 5 minutes to complete. 

The BDI-II consists of 21 items ranking intensity of symptoms on a scale of 0–3, with 3 indicating greatest severity of a particular symptom. Possible scores for total BDI range 0–63. The higher the score indicates higher level of depression, for example, minimal depression (0–13), mild depression (14–19), moderate depression (20–28), and severe depression (29–63) [21]. Based on results of past factor analytic studies, two subsets emerge identifying 5 somatic and 16 nonsomatic symptomatology [21, 43, 44]. See Table 1. The somatic symptoms on the BDI-II (questions 15, 16, 18, 20, 21) were used to measure somatization of depression [21, page 33]. High reliability and validity have been reported for the English [21, 44] and the Spanish [45] versions. 

## 8. Analysis

Using a computerized statistical program (SAS version 9.2) a university statistician calculated descriptive and other statistics. Pearson correlation coefficients were calculated to determine associations between and among (a) continuous self-report demographic variables such as income and years of education, (b) BDI-II depression scores (total, somatic, and nonsomatic), (c) mental (MCS) and physical (PCS) functioning according to SF-36vr2, (d) pain interference, and (e) pain intensity. For categorical demographic variables, the analysis of variance was used to compare means across categories. 

Posthoc multiple regression analyses were calculated to assess predictors of depression. Due to potential for multicollinearity, we used summary variables for pain intensity and pain interference. That is, the mean of worse, least, average, and current pain was used for pain intensity, and the mean of interference on seven domains of function (question 9g on BPI) was used for pain interference. Only data from participants who had complete data for variables under study were included (*n* = 65 for demographic and functioning; *n* = 77 for pain intensity, interference, and relief). In the models for regression analysis of depression we included (a) demographic variables of gender, place of birth, marital status, educational level, and age; (b) mental and physical function variables from SF-36vr2; (c) pain variables from BPI, that is, pain intensity, pain interference, and pain relief. In addition, we conducted a logistic regression analysis of suicidal thoughts (BDI-II question 9) with the demographic, function, and pain variables listed previously. Observations decreased from 92 to 66 due to missing data. 

## 9. Results

### 9.1. Demographic Data

We recruited 92 individuals reflective of the larger southwestern community along the USA-Mexico border. The participants were predominately women (60%), Hispanic (79%), and married (60%). On average, the sample was middle-aged (*M* = 48.8 years, SD 14.7) and had median and mode annual earnings of $30,000 and $18,000, respectively. The majority of participants reported living in the USA (93%) or in the community (75%) for over 10 years. Sixty-nine participants (85%) received all or part of education in the USA with median and mean value at the 12th grade level (SD 3.7). Of interest, mean body mass index (BMI) for this sample was 29.5 (SD 6.2). Select demographics are reported in Table 2. 

Participants experienced a wide variety of neuropathic and musculoskeletal pain syndromes including arthritis, radiculopathy, chronic regional pain syndrome, fibromyalgia, and neuritis of median, ulnar, or other nerves. Pain management included a multimodal approach tailored to the individual based on recommended best practices [46, 47]. As part of standard practice, all participants were encouraged to improve physical conditioning by staying active and eating healthy. For example, participants were asked to keep a diary of walking or activity and to bring diary to each visit. Out of 90 (no treatment information for two participants) patients, data pulled from clinical records indicated that 84 were treated with one or more medications such as opioid/nonopioid analgesics or adjuvant medications such as antidepressants, antiepileptic drugs (AED), and sleep aids (hypnotics, low dose tri- and tetracyclic antidepressants, and low dose benzodiazepines) or a combination. Antidepressant medication was used by over a third of the participants (*n* = 33) including three individuals with a prior diagnosis of depression—one with suicidal thoughts and two with comorbid anxiety. Curiously out of the same 90 patients, only 79 individuals reported they were on a treatment plan. The other 11 were treated symptomatically with nonpharmacologic treatment or topical or oral nonopioid analgesics with or without an AED. 

### 9.2. Pain

On average, participants experienced moderate (average, least, worst, and current) pain intensity throughout the past 24 hours (see Table 3). According to aggregate responses on the BPI, pain interfered moderately with work, general activity, enjoyment of life, mood, sleep, relations with other people, and walking ability. On average participants noted that medications and other pain treatments relieved pain about 46% (SD = 29) in the past 24 hours.

### 9.3. Depression and Suicidal Thoughts

Although the mean BDI-II score suggests that the sample was mildly depressed (*M* = 14.5, SD = 11.5), over 40% (*n* = 42) had significant depression, that is, scores indicating mild-to-severe depression. Of total sample (*n* = 92), 22% (*n* = 20) reported suicidal thoughts or wishes including two participants who reported desire to kill self or would kill self if had the chance. Three participants with previous diagnosis of depression had scores reflecting severe depression and reported thoughts of harm despite antidepressant therapy. As expected the percentage of participants with suicidal ideation increase with severity of depression. No clear trends emerge when comparing participants who were depressed, expressed suicidal thoughts, and had documented antidepressant therapy (see Table 4). 

### 9.4. Somatic and Nonsomatic Symptoms of Depression

Over half of the participants experienced all five somatic symptoms of depression: 85% participants experienced loss of energy, 82% change in sleeping patterns, 71% tiredness or fatigue, 61% concentration difficulty, 56% changes in appetite, and 54% loss of interest in sex. The most problematic nonsomatic symptoms reported were loss of pleasure (66%) and agitation (51%). 

### 9.5. Function

As previously mentioned, pain interfered with all seven domains of function listed on the BPI. Primarily, pain interfered with work, general activity, and enjoyment of life (mean scores of 5.0–5.2) and frequently with mood, sleep, relations with other people, and walking ability (mean scores of 3.8–4.6). According to SF-36vr2 results, over the previous four weeks participants' physical and emotional functions were strikingly lower than that of the average American. Mean summary scores on the SF-36vr2 were 39.8 (CI = 35.7–42.8) for physical functioning and 34.7 (CI = 31.7–37.7) for mental functioning. As previously mentioned, with norm-based scoring the expected score for an average American is 50.

### 9.6. Associations

Gender/sex, country of birth, age, ethnicity, and years living in the USA were not associated with pain, function, depression, or depressive symptoms. Annual household income was negatively correlated with total depression (*r* = −.27, *P* = .023), nonsomatic depressive symptoms (*r* = −.28, *P* = .016), and pain intensity (worse, *r* = −.25, *P* = .035; average, *r* = −.27, *P* = .02). No significant correlation was found between income and somatic symptoms or functioning, physical or mental. Education was not associated with depression or pain intensity but correlated negatively with income (*r* = −.32, *P* = .007) and physical functioning (*r* = −.28, *P* = .024). BMI was associated with worst pain levels (*r* = 0.243, *P* < .05).

For the most part, overall depression scores correlated negatively with mental/physical functioning and positively with pain intensity. The association between somatic symptoms and physical functioning was negative and nonsignificant. Conversely, there were significant negative associations between nonsomatic symptoms and both mental and physical functioning.  Table 5 captures noteworthy correlations; statistically significant *r* values are bolded.

As expected, total depression correlated with somatic (*r* = 0.86, *P* < .01) and nonsomatic (*r* = 0.98, *P* < .01) depression scores, and somatic and nonsomatic symptom scores correlated with one another (*r* = 0.76, *P* < .01). Mental and physical functioning, that is, summary physical and mental composite scores, correlated significantly with one another (*r* = 0.61, *P* < .0001). 

Pain intensity correlated for current, worse, least, and average pain in 24 hours (*P* < .0001) but was not associated with pain relief. Pain interference variables correlated significantly with one another (*P* < .0001). Due to potential for multicollinearity, summary variables for pain intensity and pain interference were used in post hoc regression and bivariate analysis.

### 9.7. Regression and Bivariate Analysis

Predictors of depression (BDI-II total) were mental functioning (*P* < .0001) and pain interference (*P* = .04). As expected, mental functioning (*P* < .0001) predicted nonsomatic symptomatology, and physical functioning (*P* < .006) predicted somatic symptomatology. Pain intensity, pain relief, and demographic variables were not predictive of depression scores or somatic/nonsomatic symptomatology. Probability of suicidal thoughts increased with decreased mental functioning. See Table 6 for odds ratio estimates.

## 10. Discussion

Gender and ethnicity did not influence expression of depression; that is, somatization of depression was not found. Hispanic and non-Hispanic men and women with chronic pain presented with both somatic (physical) and nonsomatic (emotional) symptoms of depression.

Despite pain management based on best practices, socioeconomic position influenced outcomes. The lower the income, the more likely the individual suffered higher pain intensity and depression. 

Like previous studies [4, 24, 25], participants with comorbid pain and depression experienced decreased functioning. As level of depression increased, level of pain increased and level of functioning decreased. So much so that almost a quarter of the participants reported moderate-to-severe depression, and another quarter of the participants reported suicidal ideation.

Unlike other published reports, we used a sample of chronic pain patients who received individualized, multimodal pain treatment that included antidepressant therapy. Compared to our previous work on psychological distress and pain in a similar population, pain intensity (severe versus moderate) and suicidal ideation (33% versus 22%) were lower in this study [48]. A plausible explanation is the use of antidepressants. Antidepressant therapy for individuals suffering from comorbid pain and depression seems to improve outcomes [49]. 

Group means do not always reflect actual picture. Although the mean depression score suggested mild depression, individual scores placed several participants at risk for significant depression. In addition, overall depression score, antidepressant therapy, or pain intensity did not predict suicidal thoughts or intent. Only mental functioning as measured by the SF-36v2 was predictive. However, the SF-36vr2 is proprietary and requires the purchase of special software to score, limiting its usefulness for research or practice in clinical settings. The BDI-II is proprietary, also, limiting its usefulness in clinical settings. 

Further research is needed to determine the best way to identify and treat chronic pain sufferers with depression and to monitor suicidal ideation in chronic pain population. Moreover, in studies of chronic pain and depression, researchers need to be aware that the average depression score may not reflect the risk of depression or presence of suicidal intent. A reasonable alternative is the use of categorical level of depression (minimal, mild, moderate, severe) for predictor identification.

A potential confounding variable that impacts functioning in a chronic pain population is obesity. Outcomes tend to be worse for those individuals who are overweight or obese [50]. Obesity is diagnosed in individuals with a BMI of 30. Our sample had an average BMI of 29.5, reflective of the growing problem of obesity in Hispanics residing along the border. Because chronic pain interferes with activity, sufferers are at increased risk for obesity; in turn, obesity leads to increased pain and creates a vicious cycle. Therefore, we recommend treating BMI as a confounding variable in future research on pain and depression. 

## 11. Study Limitations

The greatest limitation of this study is the loss of data. Participants were least likely to complete information about income (17) and education (9) and to answer all questions on the SF-36vr2 survey (15 missed answering one or more questions). A few skipped questions on BPI related to pain interference (4) and on BDI-II (2-3), in particular, questions about suicidal thoughts, past failure, punishment feelings, and worthlessness. When conducting grouped associations or regressions, this limited our sample size, sometimes by as much as 28 observations. In these cases, because of insufficient power, we might have missed significant associations or possible predictive variables. Conversely, the significant findings found remain significant, clinically and statistically. 

The survey was distributed in a busy medical practice. Due to time constraints or insufficient training, staff did not review the completeness of the surveys prior to participants leaving the clinic. An alternative explanation is that the survey was long (97 questions) and time consuming (30–40 minutes). Individuals may have missed questions or purposively not answered. For example, residents along the border may be hesitant to report income because of nationalization status or seasonal work. Other participants may find questions threatening, such as the one participant who scored in the severe depression category on the BDI-II and failed to answer questions about suicidal thoughts and past failures. 

Solutions to incomplete data include statistical manipulation, better training, and fidelity assessment of clinic staff, or the hiring of a research assistant who checks for completeness of data while participant is in the clinic. The first may cause incorrect interpretations of the data, the second may not be feasible in a busy or small practice, and the last is costly. Another solution is the use of flexible, short, reliable, and valid surveys that measure patient-reported health status, like the PROMIS Surveys. Funded by the NIH, PROMIS stands for Patient Reported Outcomes Measurement Information System (PROMIS). The service is free to researchers and clinicians alike. For more information see PROMIS web site at http://nihpromis.org/?AspxAutoDetectCookieSupport=1. 

Some readers may question the pooling of chronic pain patients with different diagnoses. Chronic conditions may contribute to the pain experience. However, chronic pain has a pathophysiology of its own. Although different pain-related conditions may require specific treatment for the underlying cause, the management of chronic pain is similar [22]. Moreover, there is evidence demonstrating lack of association between depression and pain types or sites [28], thus, supporting the pooling of chronic pain participants for this study.

## 12. Conclusions

Regardless of gender or ethnicity, persons with chronic pain will disclose symptoms of depression. Using depression screening tools in stigmatized conditions may be particularly important in order to identify individuals at risk for suicide. Followup on question of suicidal thoughts or intent is critical to overall well-being.

It is easy for the provider to dismiss physical symptoms and decreased psychosocial function as pain related, when they may be due to depression. Because (a) comorbid depression impacts pain and function [16], (b) antidepressant therapy is an effective adjuvant treatment for chronic pain [47], and (c) treatment of depression improves mental function and limits interference of pain on work and daily activities [48], screening for depression is essential for best practices in chronic pain [46]. Followup on symptoms of depression and function are key to quality pain management.

Tailoring pain care and promoting self-management are two ways to improve pain outcomes [22]. For chronic pain population this means assessing depression and function at each clinical encounter and developing a treatment plan with the individual that includes self-monitoring and active involvement in pain management. 

## Figures and Tables

**Table 1:** BDI-II somatic and nonsomatic depressive symptomatology [21].

Somatic symptoms	Non-somatic symptoms	
Loss of energy	Sadness	Self-criticalness
Changes in sleeping pattern	Pessimism	Crying
Changes in appetite	Past failure	Agitation
Tiredness or fatigue	Loss of pleasure	Loss of interest
Loss of interest in sex	Guilty feelings	Indecisiveness
	Punishment feelings	Worthlessness
	Self-dislike	Irritability
	Suicidal thoughts or	Concentration
	wishes	difficulty

**Table 2:** Select demographics.

		*n *	**%**
Gender (*n* = 92)	Women	55	60%
	Men	37	40%
Ethnicity (*n* = 92)	Hispanic	73	79%
	Non-Hispanic	19	31%
Marital status (*n* = 92)	Married	55	60%
	Not married	37	40%
Country of birth (*n* = 92)	USA	59	64%
	Mexico	31	34%
	Other	2	2%
Years in the USA (*n* = 90)	<10 yrs	6	7%
	>10 yrs	84	93%

**Table 3:** Current and worst, least, and average pain intensity in past 24 hours.

	*n*	Mean	SD	Range
Worst pain in 24 hours	91	5.9	2.9	0–10
Least pain in 24 hours	91	4.0	2.7	0–10
Average pain in 24 hours	90	4.8	2.6	0–10
Current pain	89	4.6	2.9	0–10

**Table 4:** Comparison of number of participants with depression, number taking antidepressants, number with suicidal thoughts, and number with suicidal thoughts and taking antidepressants (*n *= 92).

Level of Depression (BDI-II)	Number of participants taking antidepressants	Number with suicidal thoughts	Number with suicidal thoughts and taking antidepressants
Severe (*n* = 13)	5 (38.5%)	9∗ (69.2%)	3 (23.0%)
Moderate (*n* = 8)	3 (37.5%)	3 (37.5%)	4 (50.0%)
Mild (*n* = 21)	9 (42.9%)	4∗ (19.1%)	0 (0.0%)
Minimal (*n* = 50)	16 (32.0%)	4 (8.0%)	1 (2.0%)

^*^Includes one participant who reported “would kill myself if I had the chance.”

**Table 5:** Associations between depression, non-somatic/somatic depressive symptom scores, mental/physical functioning, and pain intensity.

		BDI-II Total	Non-somatic Symptoms	Somatic Symptoms
Mental functioning (MCS)	Pearson *r*	−**.278**	−**.291**	−.202
	Sig (2-tail)	**.019**	**.014**	.091
	*n *	71	71	71
Physical functioning (PCS)	Pearson *r*	−**.302**	−**.335**	−.153
	Sig (2-tail)	.010	**.004**	.203
	*n *	71	71	71
Worst pain in 24 hours	Pearson *r*	**.326**	**.292**	**.361**
	Sig (2-tail)	.002	.005	.000
	*n *	90	90	90
Least pain in 24 hours	Pearson *r*	.200	.186	.202
	Sig (2-tail)	.059	.079	.056
	*n *	90	90	90
Average pain in 24 hours	Pearson *r*	**.289**	**.267**	**.296**
	Sig (2-tail)	.006	.011	.005
	*n *	90	90	90
Current pain	Pearson *r*	**.259**	**.229**	**.294**
	Sig (2-tail)	.014	.031	.005
	*n *	89	89	89

**Table 6:** Oddsratioestimates for suicidal thoughts.

Effect	Point estimate	95%Wald confidencelimits	
Gender	1.65	0.39	6.92
Place of birth	0.08	0.00	1.69
Marital status	1.007	0.23	4.42
Education	0.34	0.02	5.02
Age	1.00	0.95	1.07
Mental functioning (MCS)	**0.91**	**0.84**	**0.98**
Pain interference	1.03	0.68	1.56
Pain intensity	1.13	0.71	1.80
